# Gout and Rheumatism—Their Pathology and Treatment

**Published:** 1845-11

**Authors:** 


*Gout and Rheumatism—their Pathology and Treatment.*—Dr. Bence Jones's treatise,* in which he endeavors to apply the physiological and chemical doctrines of Liebig to the elucidation of the pathology and therapeutics of gravel, calculus, and gout, has already been noticed in this Journal. Dr. Todd,† though admitting the humorial origin of gout and rheumatism,

* On Gravel, Cuculus, &c., by H. Bence Jones, M. B.; London, 1842.
† Croonian Lectures, by R. B. Todd, M. D., 8vo. 1843.

denies that lithic acid is the materies morbi in gout, which must, he thinks, be looked for as a compound derived from the unhealthy action of the stomach and duodenum, and which being taken into the blood, unites with elements of bile that have accumulated there, through defective secretory action of the liver. The copious deposits of lithic acid often observed in the urine for weeks or months without the occurrence of gout, he thinks sufficiently prove that lithic acid cannot be the materies morbi, and in like manner he infers, from the non-existence of lithic acid in excess, in the urine in certain cases of gout, "that the morbid element of the disease may be present independently of lithic acid;" and he particularly insists that low, depressed states of the system are favorable to the development of the gouty paroxysm. Rheumatism he believes to consist in the presence of the same morbid element (lactic acid) in the blood, and calls attention to the important fact that the rheumatic diathesis may exist without presenting the usual phenomena of rheumatism, and that in this condition the heart may become seriously affected. The cardiac inflammation may in fact be primary, and when co-existing with the articular affection, is not usually to be viewed as the result of metastasis. He devotes a chapter to the connection of rheumatism and uterine derangement, and adduces importtant reasons for believing that the accumulation of rheumatic matter in the blood may be the result of defective uterine action.

M. Briquet having employed with advantage sulphate of quinine in the treatment of typhoid fever, has had recourse to it in acute rheumatism. In his memoir, read to the French Academy,* he has detailed 23 cases treated in the following heroic manner: On the first day, 4, 5, or 6 grammes (ʒj to ʒiss.) of the sulph. quinæ (according to the age, &c., of the patient) were given, suspended in mucilage, in divided doses in the course of twelve hours. The same doses were repeated on the second and third days, when the symptoms had usually abated, and the doses were gradually diminished by grs. xx. per diem. The average duration of the pain and swelling of the joints was from three to five days. In more than one third there was cardiac complication, recent or chronic. In all but four there was a marked abatement of the symptoms in twenty-four hours. The date of the affection did not influence the cure. Relapses occurred in two only. M. Devergie, in testing Briquet's statements† began with smaller doses, and gradually increased them, and made trial of the same remedy in chronic cases. He confirms Briquet's views, except that in acute cases he would give

---

* é Sance, Oct. 15, 1842; Gazette des Hôpitaux, Nov. 17.
† Gazette Médicale, Dec. 30, 1842.

smaller doses than in the chronic. Other examples of the efficacy of Briquet's plan may be found scattered through the French journals, and Signor Mascheroni treated 40 cases in the Lodi hospital* with the best results, two or three only presenting any cardiac affection. The general result, however, of the investigations to which Briquet's memoir has led, is decidedly opposed to both the safety and utility of his plan. Several fatal cases have occurred in the French hospitals† from these heroic doses. The conflicting opinions in reference to the toxical effects of large doses of quinine induced M. Melier to investigate the whole subject afresh, and Messrs. Andral, Beguin, &c., have reported on the memoir presented by Melier to the French Academy.‡ His experiments sufficiently prove the poisonous effects on dogs, of large doses, viz: gr. 15 and upwards. The blood was always found fluid, and the brain, lungs, and gastro-enteric mucous membrane, congested. The symptoms in men and dogs are similar, viz: intoxication, disturbance of the senses, diarrhœa, hæmaturia, amaurosis, deafness, (very frequent) aphonia, delirium, coma, epileptiform, convulsions, and death. [These statements correspond with those of Giaccomiri, as the result of his experiments; "Annali Univers. di Medicina." March, 1841.] Melier shows that the utility of moderate doses of quinine in certain forms of rheumatism had been long ago pointed out by other physicians, e. g., Morton, Leroy, &c., and the reporters refer to Haygarth's clinical researches, who obtained the best results from doses of gr. 10 and upwards of *bark* every four hours. Dr. Popham's observations on this subject§ induce him to believe that bark is most useful in the fibrous form of rheumatism, and after the more acute symptoms have been combated by antiphlogistic means. If cardiac symptoms are present, the bark should be deferred until these are overcome. Periodicity of the symptoms, whether produced by the treatment or peculiar to the attack, calls for bark, and especially when profuse colliquative acid sweats are present, and the pulse small and feeble. Dr. J. J. Furnival‖ contends that acute rheumatism consists essentially in an acid state of the blood, and that the best treatment consists of the use of alkalies and antiphlogistics, since adopting which, he has never met with a single example of cardiac complication. The treatment of rheumatism by large doses of nitre has also attracted much attention, M. Martin Solon¶ appears to have been led to this mode of treatment by the observations of

* Gazetta Medica di Milano, Feb 1843.

† L'Examinateur Mèdicale, t. iii., No. 16; and Gaz. des Hôp. 11 April, 1843.

‡ Bulletin de l'Acad. Roy. de Méd., 31 May and 15 June, 1843.

§ Dublin Medical Journal, Sept. 1844.

‖ Lancet, June 1, 1844.

¶ Bull. de l'Acad. Roy., &c., t. ix. p. 130. See also, for further observations on nitre treatment, Allgem Med. Cent. Zeitung, 25 Mar. 1843, par Dr. C. F. Bartels.

Brocklesby, Macbride, and others, and by the consideration of the contra-stimulant, temperant qualities of the salt. Since 1840 he has thus treated 33 cases of severe acute rheumatism, demanding active means, of which 20 were cured from the second to the seventh day of treatment. Nitre, he states, is easily tolerated by rheumatic patients in doses of from ℥v. to ℥xv., in the 24 hours, if given in large quantities of diluent drinks. It is in acute cases only that it is useful, and its sole apparent effects are diminution of the heat of the skin, and of the frequency of the pulse. It prevents the occurrence of endocarditis, and shortens the period of convalescence; but in complicated cases does not supersede the necessity for blood-letting. M. Monneret,* however, in an instructive memoir on the comparative effects of treatment by colchicum, nitre, and blood-letting, states that the influence of nitre on the progress of eight severe cases appeared abosolutely null. Neither the heat of the skin nor quickness of the pulse was in the least affected. Professor Forget,† on the contrary, contends that nitre in large doses is a remedy of real efficacy *in certain cases*, and that in doses of from 8 to 45 drachms, given with diluents, it is rarely productive of any ill consequences. M. Requin's experiments‡ are strongly corroborative of the efficacy of Dr. Corrigan's treatment by opium, but do not justify the abandonment of depletion.—*Brit. and For. Med. Rev.*, in *Bull. of Med. Science.*

* Arch. Gén. de Méd., 1844. p. 259.

† Bull. Gén. de Thérap., t. xxv., p. 5.

‡ Bull. l'Acad., Oct. 1843.

